# Early change in circulating tumor DNA as a potential predictor of response to chemotherapy in patients with metastatic colorectal cancer

**DOI:** 10.1038/s41598-019-53711-3

**Published:** 2019-11-22

**Authors:** Hiroki Osumi, Eiji Shinozaki, Kensei Yamaguchi, Hitoshi Zembutsu

**Affiliations:** 1 Department of Gastroenterology, Cancer Institute Hospital, Japanese Foundation for Cancer Research, Tokyo, Japan; 2 Cancer Precision Medicine Center, Research Institute, Japanese Foundation for Cancer Research, Tokyo, Japan

**Keywords:** Cancer, Chemical biology, Genetics, Oncology

## Abstract

The impact of ctDNA changes after chemotherapy on the clinical outcomes of patients with metastatic colorectal cancer (mCRC) remains unclear. The present study evaluated the clinical implications of the early change in ctDNA levels as a predictor of objective response and clinical outcome in mCRC patients who received chemotherapy. We investigated the effects of after/before ratio of ctDNA levels 2 and 8 weeks after initiation of second-line chemotherapy, on objective response rate (ORR), progression-free survival (PFS), and overall survival (OS). ctDNA was detected using amplicon-based deep sequencing with a molecular barcode encompassing >240 hotspot mutations in 14 colon cancer-related genes. In multivariate analysis, as compared to baseline, patients with lower ctDNA level (≤50%) 8 weeks after initiation of chemotherapy showed significantly longer PFS and OS than the patients with higher (>50%) ctDNA level. In patients achieving a partial response or stable disease, the after/before ratio of ctDNA level 8 weeks after initiation of chemotherapy was significantly lower than those in patients with progressive disease. The present study suggests that an early change in the ctDNA level might serve as a biomarker to predict the chemotherapeutic efficacy and clinical outcomes in patients with mCRC.

## Introduction

Liquid biopsy is a non-invasive method to detect tumor-related mutations in the plasma of patients with cancer^1–3^. It enables analyses of circulating tumor cells, circulating cell-free DNA (cfDNA), and the exosomes secreted from cancer cells^4–6^. In comparison to tissue biopsy, liquid biopsy involves minimal procedural risk for the patients and is less expensive^1,5^. Furthermore, it facilitates early diagnosis of cancer and detection of tumor recurrence, serves as a substitute for tissue examination, and enables effective monitoring of the early effect of chemotherapy and drug resistance^5,6^. As a result of the recent advancement in sequencing technologies that detect and quantify cancer-related genomic variants in cfDNA, studies on circulating tumor DNA (ctDNA) are rapidly increasing^7,8^. cfDNA is released from healthy, inflamed, and cancerous tissues undergoing apoptosis or necrosis^2,5^. ctDNA is a small fraction of cfDNA that originates from the tumor cells, generally characterized by the presence of somatic variants^2,5^. The Food and Drug Administration approved the cobas epidermal growth factor receptor (EGFR) Mutation Test v2 to identify the T790M mutation in the plasma of patients with non-small cell lung cancer^9,10^. However, there is no reliable genomic testing to guide the treatment decisions for patients with metastatic colorectal cancer (mCRC) in current clinical practice^11–13^. Several studies reported *RAS* mutations in cfDNA using digital PCR (dPCR) and its possible clinical implications in patients with colon cancer^14–16^. Previous studies indicated that the amplicon-based next-generation sequencing (NGS) with molecular barcode detect multiple mutations in the plasma maintaining the sensitivity comparable to dPCR^17,18^. It was also reported that ctDNA levels change during chemotherapy and the increase was noted prior to the elevation of the tumor marker levels or disease progression as confirmed by computed tomography (CT)^5,19^. However, only a few studies spotted the correlation between early changes in ctDNA, (which were detected by deep sequencing method using the amplicon-based NGS with molecular barcode) and survival in mCRC patients who underwent chemotherapy^20,21^. Moreover, because the response rate of second-line chemotherapy is likely to be lower than those of first-line chemotherapy, the development of a new surrogate marker for clinical response (survival) after second-line chemotherapy other than tumor shrinkage is important to provide mCRC patients with effective second-line chemotherapy. In the present study, we aimed to investigate the correlation between early response of ctDNA and clinical response after chemotherapy in mCRC patients using a deep-sequencing system with NGS and evaluated the ctDNA response which might ease the clinical decision-making process.

## Results

### Patient characteristics

To detect the ctDNA in plasma, we recruited 29 mCRC patients receiving second-line chemotherapy. The characteristics of these 29 patients with mCRC are summarized in Table 1. Their median age at the time of recruitment was 57 years (range, 39–76 years). Of the 29 patients, 14 were males (48.3%). The liver was the most frequent site of metastasis (93.1%), followed by the lung (48.3%), peritoneum (31.0%), and lymph node (24.1%). Ten patients (34.5%) harbored wild-type RAS in their tumor tissues, and out of them, 6 (20.7%) patients received anti-EGFR antibody therapy before blood sample collection (Table 1).

**Table 1:** Patient demographics and clinical characteristics.

Characteristics	Total (N = 29) No. of patients (%)
Age at enrollment, years	
Median [range]	57 [39-76]
Gender	
Male	14 (48.3)
Female	15 (51.7)
Treatment line at the time of sampling	
FOLFIRI + bevacizumab	15 (51.7)
FOLFIRI + ramucirumab	10 (34.5)
FOLFOX + bevacizumab	4 (13.8)
Primary site	
Right-sided colon	13 (44.8)
Left-sided colon	16 (55.2)
Resection of primary tumor	
Yes	18 (62.1)
No	11 (37.9)
Metastatic site	
Single organ	8 (27.6)
Multi-organ	21 (72.4)
Liver	27 (93.1)
Lung	14 (48.3)
Peritoneal	9 (31.0)
Lymph node	7 (24.1)
Other	3 (10.3)
*RAS* status in tissue	
Wild type	10 (34.5)
Mutant	19 (65.5)
Prior Chemotherapy regimen	
Anti-VEGF antibody	21 (72.4)
Anti-EGFR antibody	6 (20.7)
Cytotoxic drug(s) only	2 (6.9)
Tumor markers (at initiation of second-line chemotherapy)	
CEA median, [range]	48.6 [3.4–1119.9]
CA19-9 median, [range]	62.1 [2.0–8017.7]

FOLFIRI:a combination of leucovorin and fluorouracil with irinotecan.

VEGF:vascular endothelial growth factor.

FOLFOX:a combination of leucovorin and fluorouracil with oxaliplatin.

EGFR:epidermal growth factor receptor.

5-FU:5-fluorouracil.

LV:leucovorin.

*RAS*:rat sarcoma viral oncogene homolog.

CEA:carcinoembryonic antigen.

CA19-9:carbohydrate antigen 19-9.

### Detection of somatic mutations in plasma

**Figure 1:**
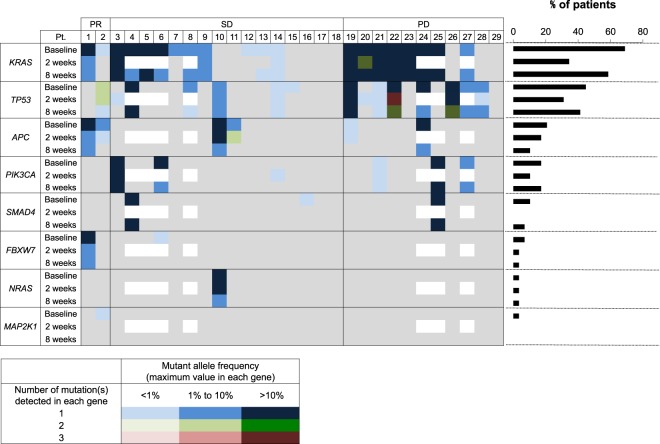
Mutant allele frequencies in cell-free DNA (cfDNA) of metastatic colorectal cancer (mCRC) patients. Genomic landscape of the mutations detected in the plasma of 29 patients with mCRC. The numbers and frequencies of the mutant alleles in 8 genes detected in 29 patients are shown. Grey panel, no mutation detected; White panel, not tested.

Of the 29 patients recruited in this study, one or more somatic mutations in the 8 colorectal cancer-related genes (*KRAS*, *TP53*, *APC*, *PIK3CA*, *SMAD4*, *FBXW7*, *NRAS*, and *MAP2K1*) were detected in 26 (89.7%) patients with mCRC, while no mutations in the remaining 6 genes (*AKT1*, *BRAF*, *CTNNB1*, *EGFR*, *ERBB2*, and *GNAS*) were detected. Mutations in *KRAS*, *TP53*, and *APC* were detected in 20 (69.0%), 13 (44.8%), and 6 (20.7%) patients at baseline, respectively (Fig. 1). *PIK3CA and SMAD4* were also frequently mutated in 5 (17.2%) and 3 (10.3%) patients, respectively (Fig. 1). Mutations in *FBXW7* (6.9%), *NRAS* (3.4%), and *MAP2K1* (3.4%) were less common (<10% of patients) compared to those in other genes (Fig. 1).

### Association between early change in ctDNA levels and clinical outcomes after second-line chemotherapy

**Figure 2:**
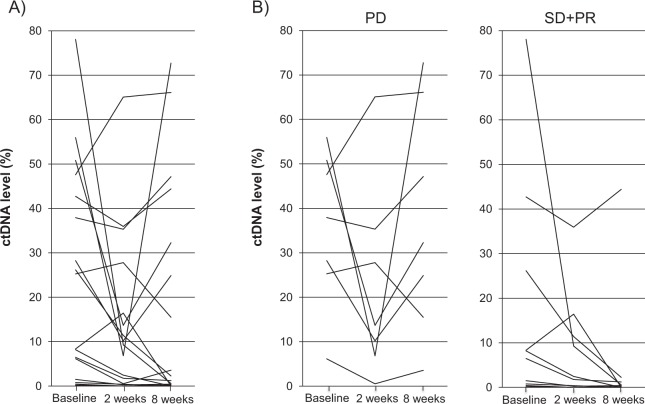
Changes in ctDNA levels in mCRC patients treated with second-line chemotherapy. ctDNA analysis at baseline, 2 weeks and 8 weeks after initiation of second-line chemotherapy in all the patients **(A)** and the patients with PD and SD or PR **(B)**. Treatment responses were evaluated by CT images.

To assess the clinical significance of the early change in ctDNA levels in the patients with mCRC after chemotherapy, we investigated the association of ctDNA levels at 2 and 8 weeks after initiation of the second-line chemotherapy with progression-free survival (PFS) and overall survival (OS). ctDNA levels at 2 weeks (median ctDNA level, 6.8%; range, 0% to 65.1%) and 8 weeks (median ctDNA level, 3.8%; range, 0% to 72.7%) after initiation of the chemotherapy were likely to be lower than in the baseline (median ctDNA level, 17.8%; range, 0.17% to 78.1%) as shown in Fig. 2A (2 weeks vs baseline; *P* = 0.09, 8 weeks vs baseline; *P* = 0.20). The changes in ctDNA levels in patients with progressive disease (PD) and partial response (PR) or stable disease (SD) before and after the chemotherapy are shown in Fig. 2B.

**Figure 3:**
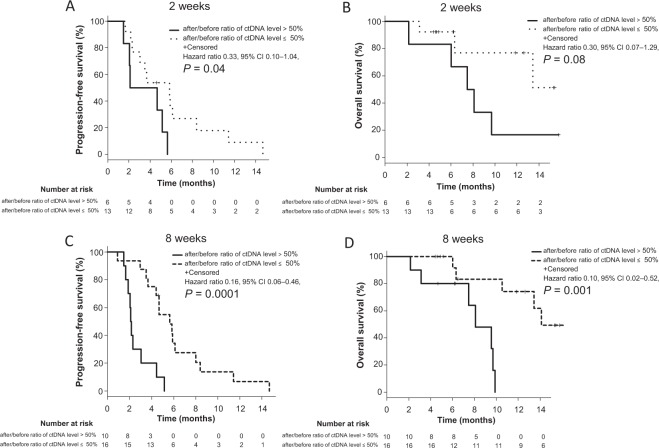
Kaplan-Meier estimates of PFS and OS with respect to ctDNA levels in mCRC patients treated with second-line chemotherapy. Comparison of PFS **(A)** and OS **(B)** in patients with after/before ratio of ctDNA level >50% and ≤50% at 2 weeks after initiation of the second-line chemotherapy. Comparison of PFS (C) and OS (D) in patients with after/before ratio of ctDNA level >50% and ≤50% 8 weeks after initiation of the second-line chemotherapy. *P* values were calculated using log-rank test.

Kaplan-Meier estimates indicated that the patients who showed ≤50% after/before ratio in their ctDNA levels 2 weeks after initiation of the chemotherapy had significantly longer PFS than those with >50% (median PFS: 5.8 vs 3.3 months; HR, 0.33; 95% CI, 0.10–1.04; *P* = 0.04, and median OS: NA vs 7.8 months; HR, 0.30; 95% CI, 0.07–1.29; *P* = 0.08, Fig. 3A,B). Similarly, patients who showed ≤50% after/before ratio in their ctDNA levels 8 weeks after initiation of the chemotherapy had significantly longer PFS and OS than those with >50% (median PFS: 5.6 vs 2.1 months; HR, 0.16; 95% CI, 0.06–0.46; *P* = 0.0001, Fig. 3C, and median OS: 14.1 vs 8.1 months; HR, 0.10; 95% CI, 0.02–0.52; *P* = 0.001; Fig. 3D). To evaluate the clinical validity of changes in absolute ctDNA counts during the second-line chemotherapy for mCRC, we further estimated Kaplan-Meier curves of PFS and OS using absolute ctDNA counts. Kaplan-Meier estimates indicated that there were no significant differences of PFS and OS between the patients who showed ≤50% after/before ratio of absolute ctDNA counts 2 weeks after initiation of the chemotherapy and those with >50% (Supplemental Fig. 1A,B). On the other hand, patients who showed ≤50% after/before ratio of absolute ctDNA counts 8 weeks after initiation of the chemotherapy had significantly longer PFS and OS than those with >50% (median PFS: 5.8 vs 2.1 months; HR, 0.18; 95% CI, 0.07–0.49; *P* = 0.0002, Supplemental Fig. 1C, and median OS: NA vs 9.6 months; HR, 0.17; 95% CI, 0.03–0.81; *P* = 0.01; Supplemental Fig. 1D). Moreover, we evaluated Kaplan-Meier curves of PFS and OS using optimal cut off value of after/before ratio of ctDNA levels calculated by the receiver operating characteristic curves. A cutoff value of 84.1% for after/before ratio in their ctDNA levels allowed the best stratification at 2 weeks, with a sensitivity of 42.9% and a specificity of 91.7% (AUC, 0.67; 95%CI, 0.41–0.93) and 39.4% at 8 weeks with a sensitivity of 90.0% and a specificity of 75.0% (AUC, 0.83; 95%CI, 0.67–0.99). Kaplan-Meier estimates indicated that the patients who showed ≤84.1% after/before ratio in their ctDNA levels 2 weeks after initiation of the chemotherapy had significantly longer PFS than those with >84.1% (median PFS: 5.6 vs 2.1 months; HR, 0.28; 95% CI, 0.09–0.95; *P* = 0.029, and median OS: NA vs 7.5 months; HR, 0.14; 95% CI, 0.03–0.72; *P* = 0.006, Supplemental Fig. 2A,B). Similarly, patients who showed ≤39.4% after/before ratio in their ctDNA levels 8 weeks after initiation of the chemotherapy had significantly longer PFS and OS than those with >39.4% (median PFS: 5.8 vs 2.1 months; HR, 0.15; 95% CI, 0.05–0.43; *P* = 0.00006, Supplemental Fig. 2C, and median OS: NA vs 9.6 months; HR, 0.10; 95% CI, 0.02–0.53; *P* = 0.001; Supplemental Fig. 2D).

In the univariate Cox proportional hazard analysis, primary tumor location, lung metastasis, changes in ctDNA level (after/before ratio of ctDNA level), CEA and CA19-9 levels (after/before ratio of CEA and CA19-9) 8 weeks after initiation of the chemotherapy were predictive factors for PFS (Table 2). Similarly, change in ctDNA levels and CA19-9 levels 8 weeks after initiation of the chemotherapy were predictive factors for OS (Table 2). In multivariate analysis, change in the ctDNA level 8 weeks after initiation of the chemotherapy was a predictive factor for both PFS (HR, 0.17; 95% CI, 0.06–0.47; *P* = 0.0006; Table 2) and OS (HR, 0.10; 95% CI, 0.02–0.52; *P* = 0.006; Table 2).

**Table 2:** Cox proportional hazard analysis for PFS and OS in mCRC patients treated with second-line chemotherapy.

PFS	Univariate analysis				Multivariate analysis			
	HR	Lower 95% CI	Upper 95% CI	*P* value	HR	Lower 95% CI	Upper 95% CI	*P* value
Gender (Female* or Male)	0.72	0.31	1.66	0.44				
Age (<65* or ≥65)	0.52	0.2	1.36	0.18				
Primary tumor location (Left* or Right)	2.42	1.04	5.62	0.04	2.1	0.86	5.13	0.1
Resection of primary tumor (No* or Yes)	0.78	0.35	1.75	0.54				
Liver metastasis (Negative* or Positive)	1.77	0.23	13.4	0.58				
Lung metastasis (Negative* or Positive)	0.32	0.13	0.8	0.02	0.4	0.14	1.1	0.08
Peritoneal metastasis (Negative* or Positive)	1.04	0.44	2.47	0.93				
Lymph node metastasis (Negative* or Positive)	1.3	0.52	3.24	0.58				
Metastatic site (1* or >1)	0.87	0.32	2.36	0.79				
Tissue *RAS* mutation (Negative* or Positive)	1.74	0.64	4.7	0.27				
Early tumor shirinkage (Negative* or Positive)	0.17	0.02	1.33	0.09				
Baseline ctDNA level (≤Average* or >Average)	1.49	0.66	3.37	0.33				
after/before ratio of ctDNA level at 2 weeks after initiation of the chemotherapy (≤50% or >50%*)	0.33	0.1	1.04	0.059				
after/before ratio of ctDNA level at 8 weeks after initiation of the chemotherapy (≤50% or >50%*)	0.16	0.06	0.46	0.0005	0.17	0.06	0.47	0.0006
after/before ratio of CEA levels at 8 weeks after initiation of the chemotherapy (≤Average or >Average*)	0.37	0.14	0.96	0.036	1.36	0.29	6.35	0.7
after/before ratio of CA19-9 levels at 8 weeks after initiation of the chemotherapy (≤Average or >Average*)	0.31	0.1	0.99	0.049	0.36	0.11	1.24	0.1
**OS**								
Gender (Female* or Male)	0.99	0.31	3.1	0.98				
Age (<65* or ≥65)	0.52	0.14	1.93	0.33				
Primary tumor location (Left* or Right)	1.4	0.44	4.4	0.57				
Resection of primary tumor (Yes* or No)	0.38	0.11	1.28	0.12				
Liver metastasis (Negative* or Positive)	2.2	0.45	10.75	0.33				
Lung metastasis (Negative* or Positive)	0.45	0.14	1.43	0.17				
Peritoneal metastasis (Negative* or Positive)	1.3	0.39	4.35	0.67				
Lymph node metastasis (Negative* or Positive)	0.45	0.1	2.1	0.3				
Metastatic site (1* or >1)	0.54	0.14	2	0.36				
Tissue *RAS* mutation (Negative* or Positive)	1.24	0.27	5.7	0.79				
Early tumor shirinkage (Negative* or Positive)	0.48	0.15	1.54	0.22				
Baseline ctDNA level (≤Average* or >Average)	2.3	0.7	7.9	0.17				
after/before ratio of ctDNA level at 2 weeks after initiation of the chemotherapy (≤50% or >50%*)	0.3	0.07	1.29	0.11				
after/before ratio of ctDNA level at 8 weeks after initiation of the chemotherapy (≤50% or >50%*)	0.1	0.02	0.52	0.006	0.1	0.02	0.52	0.006
after/before ratio of CEA levels at 8 weeks after initiation of the chemotherapy (≤Average or >Average*)	0.36	0.11	1.15	0.09				
after/before ratio of CA19-9 levels at 8 weeks after initiation of the chemotherapy (≤Average or >Average*)	0.07	0.01	0.49	0.006	0.17	0.03	1.04	0.055

*Reference

PFS:progression-free survival.

OS:overall survival.

mCRC:metastatic colorectal cancer.

HR:hazard ratio.

CI:confidence interval.

*RAS*:rat sarcoma viral oncogene homolog.

ctDNA:circulating tumor DNA.

CEA:carcinoembryonic antigen.

CA19-9:carbohydrate antigen 19-9.

### Association between early change in ctDNA after chemotherapy and therapy response

**Figure 4:**
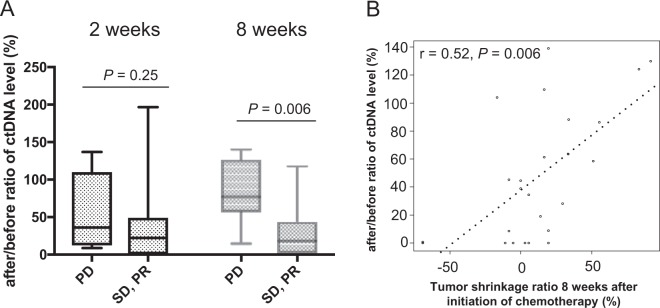
Association between ctDNA response and clinical response after chemotherapy. (**A**) ctDNA response (after/before ratios of ctDNA levels 8 weeks after initiation of the chemotherapy) is significantly associated with objective response in mCRC patients treated with second-line chemotherapy (*P* = 0.006). (**B)** ctDNA response strongly correlated with the tumor shrinkage ratio 8 weeks after initiation of the chemotherapy (r = 0.52, *P* = 0.006). *P* values were calculated by Spearman correlation method and linear regression was performed.

We next analyzed the association between objective response and change in ctDNA levels 2 weeks and 8 weeks after initiation of second-line chemotherapy in mCRC patients. Objective response rate (ORR) and disease control rate (DCR) were 6.9% and 55.2% (2 PR and 14 SD), respectively. Although there was no significant difference of change in the ctDNA levels 2 weeks after initiation of the chemotherapy between PD and SD or PR groups, patients achieving SD or PR showed significantly lower ctDNA level 8 weeks after initiation of chemotherapy compared to those with PD (2 weeks: *P* = 0.25, 8 weeks: *P* = 0.006, Fig. 4A). Furthermore, patients with ≤50% after/before ratio of ctDNA levels 8 weeks after initiation of the chemotherapy showed better objective response compared to those with >50% (*P* = 0.003, Table 3). Changes in ctDNA levels significantly correlated with tumor shrinkage 8 weeks after initiation of second-line chemotherapy (r = 0.52, *P* = 0.006, Fig. 4B). These results suggest that ctDNA could act as a prognostic biomarker to predict the outcomes of second-line chemotherapy in mCRC patients.

**Table 3:** The association between early ctDNA response and objective response.

	after/before ratio of ctDNA levels >50%	after/before ratio of ctDNA levels ≤50%	*P* value
**2 weeks after initiation of chemotherapy**			
PD	3	4	0.65
SD, PR	3	9	
**8 weeks after initiation of chemotherapy**			
PD	8	2	0.003
SD, PR	2	14	

ctDNA:circulating tumor DNA.

PD:progressive disease.

SD:stable disease.

PR:partial response.

## Discussion

The present study demonstrates a significant association between early changes in ctDNA levels and the ORR, PFS, and OS in mCRC patients treated with second-line chemotherapy. The mCRC patients whose ctDNA levels decreased up to ≤50% 2 weeks and 8 weeks after initiation of chemotherapy showed a better response to the chemotherapy. Previous reports showed that early tumor shrinkage (ETS) is pivotal early predictors of treatment efficacy in mCRC patients, especially in those treated with molecular-targeted drugs^22–24^. Here, the change in ctDNA levels at 2 weeks after initiation of chemotherapy is shown as a possible predictor of PFS (HR, 0.33; 95%CI, 0.10–1.04; *P* = 0.059, in univariate analysis; Table 2). Furthermore, the change in ctDNA level at 8 weeks after initiation of chemotherapy was an independent predictor of PFS (HR, 0.17; 95%CI, 0.06–0.47; *P* = 0.0006, in multivariate analysis; Table 2) and OS (HR, 0.10, 95%CI, 0.02–0.52; *P*** = **0.006, in multivariate analysis; Table 2). On the other hand, ETS, which is defined as the relative change in the sum of the longest diameter of the tumor at 8 weeks compared to the baseline (≥20% tumor shrinkage) in this study, was not a significant predictor of PFS (HR, 0.17; 95%CI, 0.02–1.33; *P* = 0.09, in univariate analysis; Table 2) or OS (HR, 0.48; 95%CI, 0.15–1.54; *P* = 0.22, in univariate analysis; Table 2) in our study. Our data suggest that after/before ratio of the ctDNA level after second-line chemotherapy could be a better predictor of chemotherapeutic efficacy than ETS in mCRC patients^20,25^.

Tumor markers such as CEA and CA19-9 are widely used to monitor the tumor burden and progression of mCRC during chemotherapy^26,27^. Previous reports indicated that CEA levels and survival are inversely correlated in patients receiving a combination of chemotherapy and anti-EGFR inhibitor^28,29^. In our study, univariate analysis and log-rank test showed that the mCRC patients showing reduced after/before ratio in CEA and CA19-9 levels 8 weeks after initiation of the chemotherapy was inversely correlated with PFS (CEA: HR, 0.37; 95%CI, 0.14–0.96; *P*_*univariate*_ = 0.036, *P*_log-rank_ = 0.03, CA19-9: HR, 0.31; 95%CI, 0.10–0.99; *P*_*univariate*_ = 0.049, *P*_log-rank_ = 0.04; Table 2 and Supplementary Fig. 3A,C). In addition, the mCRC patients exhibiting lower after/before ratio of CA19-9 levels 8 weeks after initiation of the chemotherapy was inversely correlated with OS (HR, 0.07; 95%CI, 0.01–0.49; *P*_*univariate*_ = 0.006, *P*_log-rank_ = 0.0005; Table 2 and Supplementary Fig. 3D). However, the change (after/before ratio) in ctDNA level at 8 weeks remained as an independent indicator of both PFS (*P* = 0.0006; Table 2) and OS (*P* = 0.006; Table 2) in multivariate analysis. Reportedly, the change in ctDNA after completion of cycle 1 of chemotherapy could be a successful predictor for response to chemotherapy while CEA failed as a predictive marker^20^. Collectively, these findings suggest that ctDNA might serve as a reliable predictive biomarker for early therapeutic response. In-depth research is warranted to further establish the critical role of ctDNA as a predictive marker for the response to chemotherapy in patients with mCRC.

The sample size in the current investigation was small. Moreover, the frequencies of mutated genes in ctDNA of patients with CRC in our study were inconsistent with those in the tissue DNA reported in the mutation database including The Cancer Genome Atlas (TCGA)^30,31^. The mutation frequency of the *APC* gene in CRC tissue has been reported to be ~80%^30,32^. However, we observed only 20.7% in plasma, which was a significantly lower frequency than those reported in tissues previously^33^. This inconsistency might be partially due to the insufficient coverage of mutations in *APC* gene that the gene panel used in this study could guarantee. Further technical improvement in the mutation detection system to detect additional mutations and gene rearrangements could increase the sensitivity for mutation detection in the plasma of mCRC patients.

In conclusion, we unraveled that in mCRC patients receiving chemotherapy, early changes in ctDNA levels represent highly sensitive early predictor of treatment response^20^. Future prospective clinical trials with large sample size should be conducted to validate the clinical impact of change in ctDNA and support its application as a novel early predictive biomarker for the response to second-line chemotherapy.

## Materials and Methods

### Patients

This study aimed to study was to study the correlation between early ctDNA response and PFS, OS, ORR in mCRC patients treated with second-line chemotherapy (Table 1). Twenty-nine mCRC patients, who were treated with second-line chemotherapies at Cancer Institute Hospital, Japanese Foundation for Cancer Research, were prospectively enrolled in this study from February 2017 to March 2018. TNM Classification of Malignant Tumors (7th edition) was used to determine the tumor and nodal status. This present study was approved by the Institutional Review Boards of the Japanese Foundation for Cancer Research (Tokyo, Japan, registry number 2017–1009). Written informed consent was obtained from all the patients for the use of their plasma and tissue samples. All methods were performed in accordance with the Declaration of Helsinki.

### Blood samples, ctDNA isolation, and sequencing

Blood samples were collected in EDTA tubes as per the manufacturer’s instructions. Time points for collecting the blood samples were just before the initiation of second-line chemotherapy and 2 weeks and 8 weeks after initiation of the second-line chemotherapy. Plasma from the blood was obtained by centrifugation at 1600 *g* for 10 min at 4 °C, followed by another spin at 16,000 *g* for 10 min at 4 °C to remove the cell debris. cfDNA was extracted from 2 mL plasma using a MagMAX cfDNA Isolation Kit (Thermo Fisher Scientific, USA) following the manufacturer’s instructions. Preparation and quality control of the libraries, template preparation, and sequencing were performed as previously described^34^. Fourteen genes with >240 hotspots (Single nucleotide variants and short indels), including *AKT1*, *BRAF*, *CTNNB1*, *EGFR*, *ERBB2*, *FBXW7*, *GNAS*, *KRAS*, *MAP2K1*, *NRAS*, *PIK3CA*, *SMAD4*, *TP53*, and *APC* were covered in this assay^34,35^. Clean reads were mapped to the human reference genome (hg19) sequence. The Torrent Variant Caller was used to filter and call the mutations in targeted regions of each gene^34,35^. The limit of detection for each variant was 0.15% in this study. ctDNA level in plasma was defined as the highest allele frequency of the detected mutant alleles at each time point in each patient when two or more mutations were detected.

### Tumor tissue DNA sequencing

Genomic DNA was extracted from fixed paraffin-embedded tissues obtained from biopsies or surgical resections as previously described^36,37^. For tissue *KRAS* and *NRAS* test, RASKET KIT (MBL, Japan), which applies the polymerase chain reaction-reverse sequence-specific oligonucleotide method (PCR-rSSO), was used following the manufacturer’s protocol. We examined twelve mutations in *RAS* exon 2, eight in *RAS* exon 3, and four in *RAS* exon 4 using Luminex 100/200 (Luminex, Japan) and UniMAG (MBL, Japan) system as described previously^38,39^.

### Statistical analyses and tumor assessment

Tumor response was assessed by CT imaging using RECIST guideline, version 1.1. PFS was defined as the time from the 1st day of the second line treatment to either the first objective evidence of disease progression or death from any cause. OS was defined as the time from the 1st day of the second line treatment until the time of death. PFS and OS were estimated using the Kaplan-Meier method and the statistical significance of the correlation between the clinical outcome and clinical parameters (ctDNA, CEA, and CA19-9 levels) was assessed using the log-rank test. ORR denotes the proportion of patients who have a complete response or PR to the second-line chemotherapy, and DCR indicates the proportion of patients who have a complete response or PR or SD to the therapy^40^. ETS is the relative change in the sum of the longest diameter of the tumor at week 8 compared to the baseline (≥20% tumor shrinkage)^24,41^. Statistical tests provided two-sided *P* values, and *P* < 0.05 was considered significant. In Cox proportional hazard analysis, factors with *P* < 0.05 in the univariate analysis were included in the multivariate analysis (Backward stepwise methods). Statistical analyses were carried out using the statistical software, “EZR” (Easy R), which is based on R and R commander^42^.

## Supplementary information

Supplemental fig.1

Supplemental fig.2

Supplemental fig.3

## Data Availability

The data that support the findings of this study are available from the corresponding author upon reasonable request.
