# Supplementary material for: Early change in circulating tumor DNA as a potential predictor of response to chemotherapy in patients with metastatic colorectal cancer
Source: Sci Rep. 2019 Nov 22;9:17358. doi: 10.1038/s41598-019-53711-3 (PMC6874682; doi:10.1038/s41598-019-53711-3)
Supplement: Supplementary file 1 — Supplemental fig.1 [file 41598_2019_53711_MOESM1_ESM.pptx]

## Slide 1
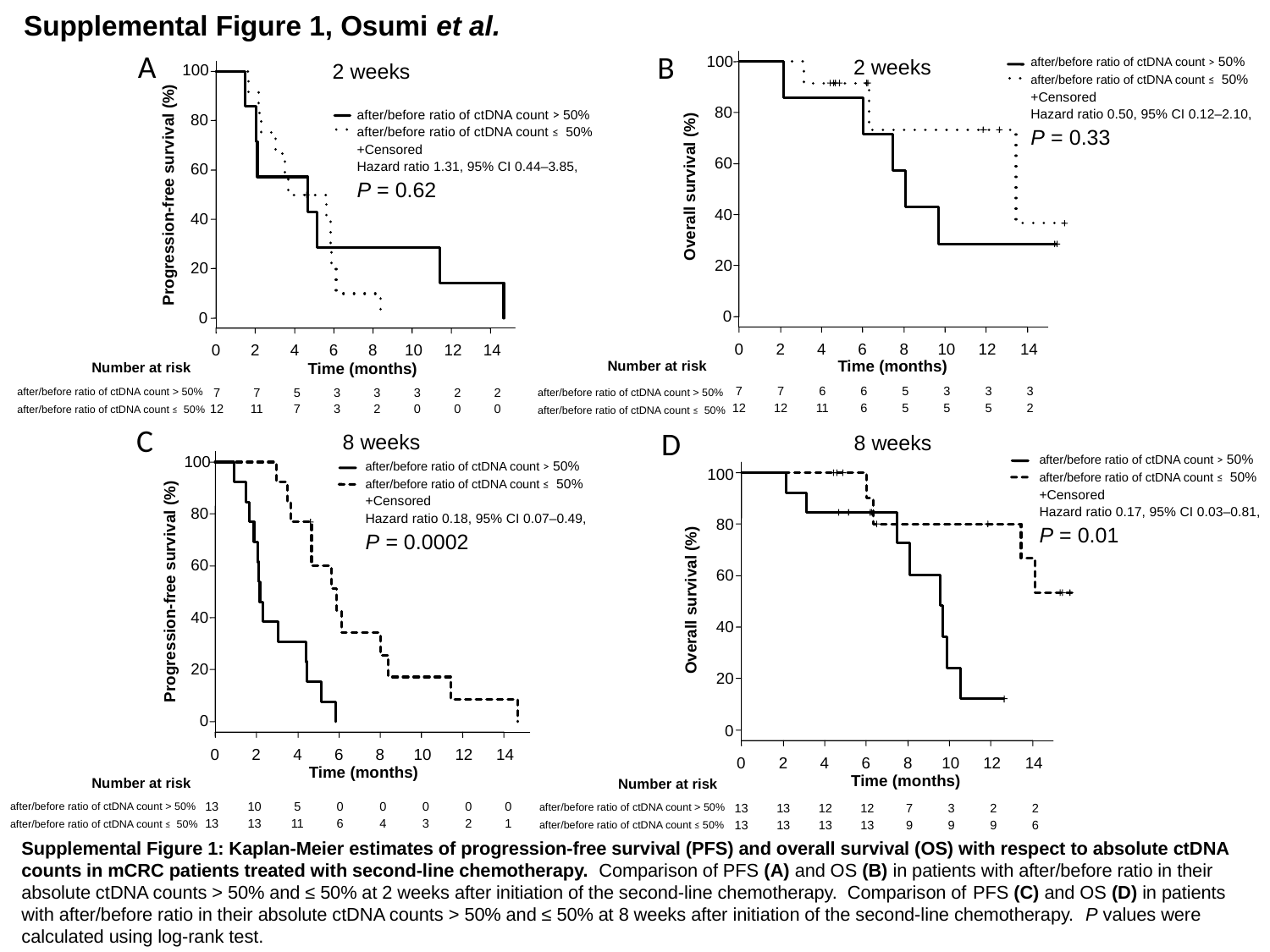

Supplemental Figure 1, Osumi et al.
A
B
after/before ratio of ctDNA count > 50%
after/before ratio of ctDNA count ≤ 50%
+Censored
Hazard ratio 0.50, 95% CI 0.12–2.10,
P = 0.33
2 weeks
100
80
60
Overall survival (%)
40
20
0
0
2
4
6
8
10
12
14
Time (months)
2 weeks
100
80
60
Progression-free survival (%)
40
20
0
0
2
4
6
8
10
12
14
Time (months)
after/before ratio of ctDNA count > 50%
after/before ratio of ctDNA count ≤ 50%
+Censored
Hazard ratio 1.31, 95% CI 0.44–3.85,
P = 0.62
Number at risk
Number at risk
after/before ratio of ctDNA count > 50%
after/before ratio of ctDNA count ≤ 50%
after/before ratio of ctDNA count > 50%
after/before ratio of ctDNA count ≤ 50%
| 7 | 7 | 6 | 6 | 5 | 3 | 3 | 3 |
| --- | --- | --- | --- | --- | --- | --- | --- |
| 12 | 12 | 11 | 6 | 5 | 5 | 5 | 2 |
| 7 | 7 | 5 | 3 | 3 | 3 | 2 | 2 |
| --- | --- | --- | --- | --- | --- | --- | --- |
| 12 | 11 | 7 | 3 | 2 | 0 | 0 | 0 |
C
D
8 weeks
8 weeks
after/before ratio of ctDNA count > 50%
after/before ratio of ctDNA count ≤ 50%
+Censored
Hazard ratio 0.17, 95% CI 0.03–0.81,
P = 0.01
after/before ratio of ctDNA count > 50%
after/before ratio of ctDNA count ≤ 50%
+Censored
Hazard ratio 0.18, 95% CI 0.07–0.49,
P = 0.0002
100
80
60
Progression-free survival (%)
40
20
0
0
2
4
6
8
10
12
14
Time (months)
100
80
60
Overall survival (%)
40
20
0
0
2
4
6
8
10
12
14
Time (months)
Number at risk
Number at risk
after/before ratio of ctDNA count > 50%
after/before ratio of ctDNA count ≤ 50%
after/before ratio of ctDNA count > 50%
after/before ratio of ctDNA count ≤ 50%
| 13 | 10 | 5 | 0 | 0 | 0 | 0 | 0 |
| --- | --- | --- | --- | --- | --- | --- | --- |
| 13 | 13 | 11 | 6 | 4 | 3 | 2 | 1 |
| 13 | 13 | 12 | 12 | 7 | 3 | 2 | 2 |
| --- | --- | --- | --- | --- | --- | --- | --- |
| 13 | 13 | 13 | 13 | 9 | 9 | 9 | 6 |
Supplemental Figure 1: Kaplan-Meier estimates of progression-free survival (PFS) and overall survival (OS) with respect to absolute ctDNA counts in mCRC patients treated with second-line chemotherapy. Comparison of PFS (A) and OS (B) in patients with after/before ratio in their absolute ctDNA counts > 50% and ≤ 50% at 2 weeks after initiation of the second-line chemotherapy. Comparison of PFS (C) and OS (D) in patients with after/before ratio in their absolute ctDNA counts > 50% and ≤ 50% at 8 weeks after initiation of the second-line chemotherapy. P values were calculated using log-rank test.
