# Supplementary material for: Early change in circulating tumor DNA as a potential predictor of response to chemotherapy in patients with metastatic colorectal cancer
Source: Sci Rep. 2019 Nov 22;9:17358. doi: 10.1038/s41598-019-53711-3 (PMC6874682; doi:10.1038/s41598-019-53711-3)
Supplement: Supplementary file 2 — Supplemental fig.2 [file 41598_2019_53711_MOESM2_ESM.pptx]

## Slide 1
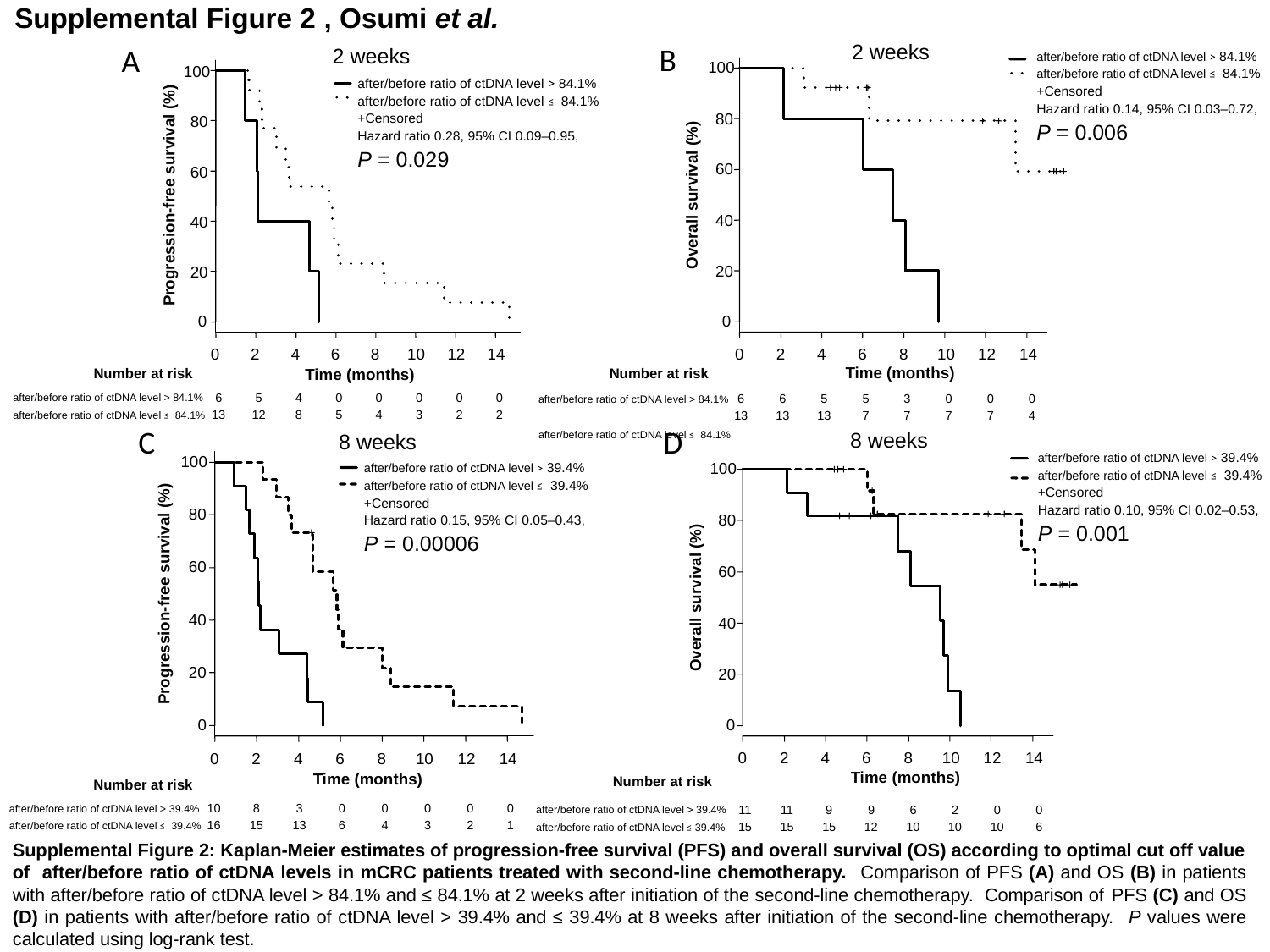

Supplemental Figure 2 , Osumi et al.
2 weeks
B
A
2 weeks
after/before ratio of ctDNA level > 84.1%
after/before ratio of ctDNA level ≤ 84.1%
+Censored
Hazard ratio 0.14, 95% CI 0.03–0.72,
P = 0.006
100
80
60
Overall survival (%)
40
20
0
0
2
4
6
8
10
12
14
Time (months)
100
80
60
Progression-free survival (%)
40
20
0
0
2
4
6
8
10
12
14
Time (months)
after/before ratio of ctDNA level > 84.1%
after/before ratio of ctDNA level ≤ 84.1%
+Censored
Hazard ratio 0.28, 95% CI 0.09–0.95,
P = 0.029
Number at risk
Number at risk
after/before ratio of ctDNA level > 84.1%
after/before ratio of ctDNA level ≤ 84.1%
after/before ratio of ctDNA level > 84.1%
after/before ratio of ctDNA level ≤ 84.1%
| 6 | 5 | 4 | 0 | 0 | 0 | 0 | 0 |
| --- | --- | --- | --- | --- | --- | --- | --- |
| 13 | 12 | 8 | 5 | 4 | 3 | 2 | 2 |
| 6 | 6 | 5 | 5 | 3 | 0 | 0 | 0 |
| --- | --- | --- | --- | --- | --- | --- | --- |
| 13 | 13 | 13 | 7 | 7 | 7 | 7 | 4 |
D
C
8 weeks
8 weeks
after/before ratio of ctDNA level > 39.4%
after/before ratio of ctDNA level ≤ 39.4%
+Censored
Hazard ratio 0.10, 95% CI 0.02–0.53,
P = 0.001
100
80
60
Progression-free survival (%)
40
20
0
0
2
4
6
8
10
12
14
Time (months)
after/before ratio of ctDNA level > 39.4%
after/before ratio of ctDNA level ≤ 39.4%
+Censored
Hazard ratio 0.15, 95% CI 0.05–0.43,
P = 0.00006
100
80
60
Overall survival (%)
40
20
0
0
2
4
6
8
10
12
14
Time (months)
Number at risk
Number at risk
after/before ratio of ctDNA level > 39.4%
after/before ratio of ctDNA level ≤ 39.4%
after/before ratio of ctDNA level > 39.4%
after/before ratio of ctDNA level ≤ 39.4%
| 10 | 8 | 3 | 0 | 0 | 0 | 0 | 0 |
| --- | --- | --- | --- | --- | --- | --- | --- |
| 16 | 15 | 13 | 6 | 4 | 3 | 2 | 1 |
| 11 | 11 | 9 | 9 | 6 | 2 | 0 | 0 |
| --- | --- | --- | --- | --- | --- | --- | --- |
| 15 | 15 | 15 | 12 | 10 | 10 | 10 | 6 |
Supplemental Figure 2: Kaplan-Meier estimates of progression-free survival (PFS) and overall survival (OS) according to optimal cut off value of after/before ratio of ctDNA levels in mCRC patients treated with second-line chemotherapy. Comparison of PFS (A) and OS (B) in patients with after/before ratio of ctDNA level > 84.1% and ≤ 84.1% at 2 weeks after initiation of the second-line chemotherapy. Comparison of PFS (C) and OS (D) in patients with after/before ratio of ctDNA level > 39.4% and ≤ 39.4% at 8 weeks after initiation of the second-line chemotherapy. P values were calculated using log-rank test.
