# Supplementary material for: Early change in circulating tumor DNA as a potential predictor of response to chemotherapy in patients with metastatic colorectal cancer
Source: Sci Rep. 2019 Nov 22;9:17358. doi: 10.1038/s41598-019-53711-3 (PMC6874682; doi:10.1038/s41598-019-53711-3)
Supplement: Supplementary file 3 — Supplemental fig.3 [file 41598_2019_53711_MOESM3_ESM.pptx]

## Slide 1
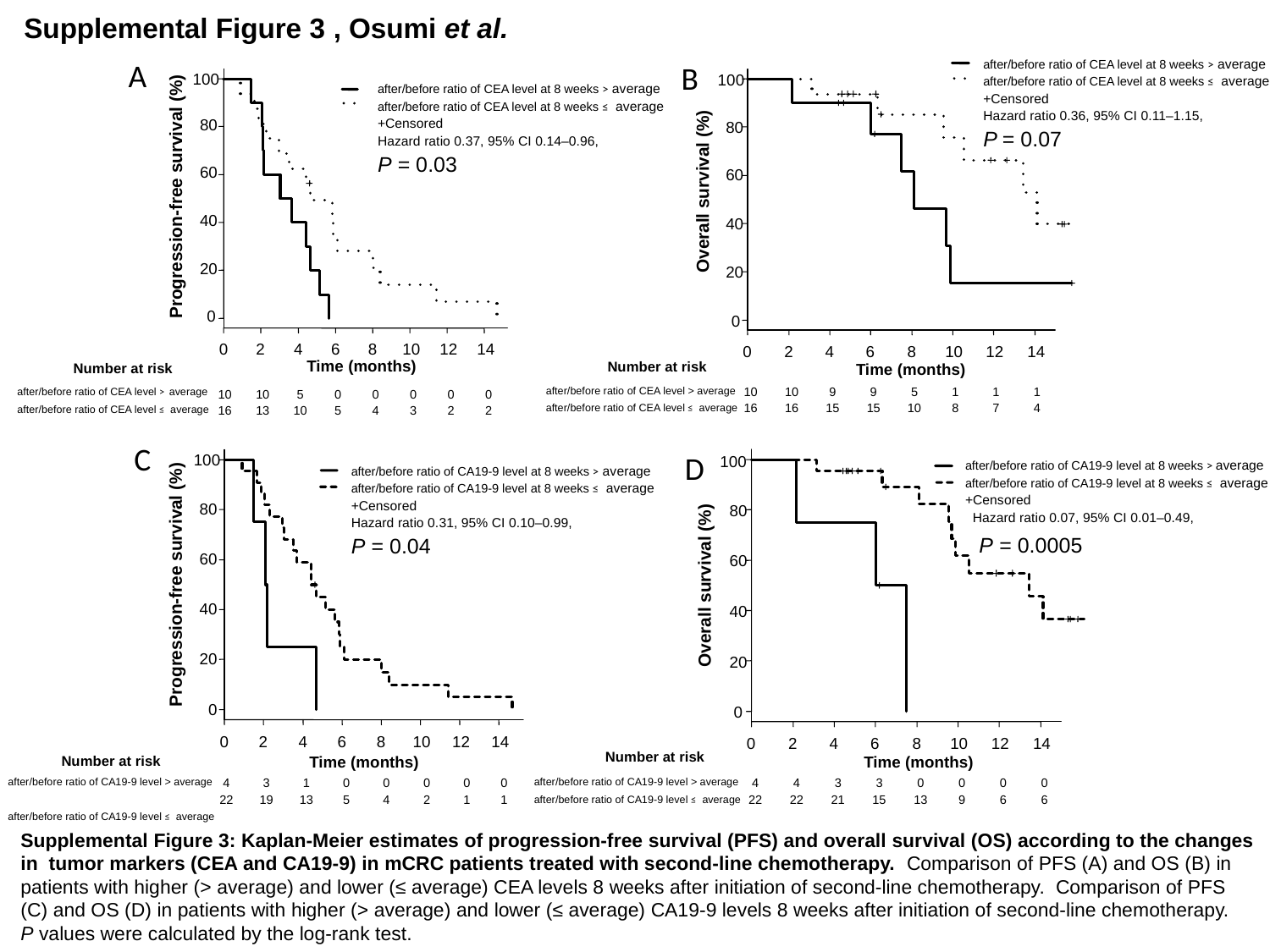

Supplemental Figure 3 , Osumi et al.
after/before ratio of CEA level at 8 weeks > average
after/before ratio of CEA level at 8 weeks ≤ average
+Censored
Hazard ratio 0.36, 95% CI 0.11–1.15,
P = 0.07
A
B
100
80
60
Overall survival (%)
40
20
0
0
2
4
6
8
10
12
14
Time (months)
100
80
60
Progression-free survival (%)
40
20
0
0
2
4
6
8
10
12
14
Time (months)
after/before ratio of CEA level at 8 weeks > average
after/before ratio of CEA level at 8 weeks ≤ average
+Censored
Hazard ratio 0.37, 95% CI 0.14–0.96,
P = 0.03
Number at risk
Number at risk
after/before ratio of CEA level > average
after/before ratio of CEA level ≤ average
after/before ratio of CEA level > average
after/before ratio of CEA level ≤ average
| 10 | 10 | 9 | 9 | 5 | 1 | 1 | 1 |
| --- | --- | --- | --- | --- | --- | --- | --- |
| 16 | 16 | 15 | 15 | 10 | 8 | 7 | 4 |
| 10 | 10 | 5 | 0 | 0 | 0 | 0 | 0 |
| --- | --- | --- | --- | --- | --- | --- | --- |
| 16 | 13 | 10 | 5 | 4 | 3 | 2 | 2 |
C
D
100
80
60
Overall survival (%)
40
20
0
0
2
4
6
8
10
12
14
Time (months)
after/before ratio of CA19-9 level at 8 weeks > average
after/before ratio of CA19-9 level at 8 weeks ≤ average
+Censored
 Hazard ratio 0.07, 95% CI 0.01–0.49,
 P = 0.0005
100
80
60
Progression-free survival (%)
40
20
0
0
2
4
6
8
10
12
14
Time (months)
after/before ratio of CA19-9 level at 8 weeks > average
after/before ratio of CA19-9 level at 8 weeks ≤ average
+Censored
Hazard ratio 0.31, 95% CI 0.10–0.99,
P = 0.04
Number at risk
Number at risk
after/before ratio of CA19-9 level > average
after/before ratio of CA19-9 level ≤ average
after/before ratio of CA19-9 level > average
after/before ratio of CA19-9 level ≤ average
| 4 | 3 | 1 | 0 | 0 | 0 | 0 | 0 |
| --- | --- | --- | --- | --- | --- | --- | --- |
| 22 | 19 | 13 | 5 | 4 | 2 | 1 | 1 |
| 4 | 4 | 3 | 3 | 0 | 0 | 0 | 0 |
| --- | --- | --- | --- | --- | --- | --- | --- |
| 22 | 22 | 21 | 15 | 13 | 9 | 6 | 6 |
Supplemental Figure 3: Kaplan-Meier estimates of progression-free survival (PFS) and overall survival (OS) according to the changes in tumor markers (CEA and CA19-9) in mCRC patients treated with second-line chemotherapy. Comparison of PFS (A) and OS (B) in patients with higher (> average) and lower (≤ average) CEA levels 8 weeks after initiation of second-line chemotherapy. Comparison of PFS (C) and OS (D) in patients with higher (> average) and lower (≤ average) CA19-9 levels 8 weeks after initiation of second-line chemotherapy. P values were calculated by the log-rank test.
